# In-patient service use before and after a mental health in-patient rehabilitation admission

**DOI:** 10.1192/bjo.2025.31

**Published:** 2025-04-01

**Authors:** Christian Dalton-Locke, Louise Marston, Justin Yang, David Osborn, Helen Killaspy

**Affiliations:** Division of Psychiatry, University College London, London, UK; Research Department of Primary Care and Population Health, University College London, UCL Medical School, London, UK; Camden and Islington NHS Foundation Trust, St Pancras Hospital, London, UK

**Keywords:** Mental health services, rehabilitation, in-patient treatment, electronic healthcare records, psychotic disorders/schizophrenia

## Abstract

**Background:**

In-patient mental health rehabilitation services provide specialist treatment to people with complex psychosis. On average, rehabilitation admissions last around a year and usually follow several years of recurrent and often lengthy psychiatric hospital admissions.

**Aims:**

To compare in-patient service use before and after an in-patient rehabilitation admission, using electronic patient healthcare records in one National Health Service Trust in London.

**Method:**

We carried out a retrospective cohort study comprised of individuals with an in-patient rehabilitation admission lasting ≥84 days between 1 January 2010 and 30 April 2019, with at least ≥365 days of records available before and after their rehabilitation admission. We used negative binomial regression models to compare the number of in-patient days before and after the rehabilitation admission.

**Results:**

A total of 172 individuals met our eligibility criteria. The median percentage of days spent as an in-patient before the rehabilitation admission was 29% (interquartile range 18–52%), and 8% (interquartile range 0–31%) after the admission. The regression model adjusted for potential confounder variables produced an incidence rate ratio of 0.520 (95% CI 0.367–0.737).

**Conclusions:**

The rate of in-patient service use was halved in the period after an in-patient rehabilitation admission compared with the period before. This suggests that in-patient rehabilitation is a clinical and cost-effective intervention in the treatment and support of people with complex psychosis.

It is estimated that around 20% of individuals who have an episode of psychosis will develop severe and complex longer-term mental health problems.^1-3^ Most have a primary diagnosis of schizophrenia, bipolar affective disorder or schizoaffective disorder, with cognitive impairments that negatively affect their motivation and organisational skills. Many will have additional problems that complicate recovery, such as pre-existing neurodevelopmental disorders (e.g. autism spectrum disorder, attention-deficit hyperactivity disorder), co-existing mental health problems (e.g. anxiety, depression, substance misuse) and/or physical health problems (e.g. cardiovascular disease, pulmonary conditions). These complex problems severely affect an individual’s ability to manage everyday tasks such as self-care, housework, shopping, cooking, budgeting and interpersonal skills, and result in high support needs.

In recent literature, people who develop these complex and longer-term mental health problems have been described as having ‘complex psychosis’. This literature includes the healthcare guideline published by the National Institute for Health and Care Excellence (NICE) specifically for this population, which recommends that all local mental healthcare systems should have a specialist rehabilitation care pathway for people with complex psychosis.^3^ This pathway should be comprised of in-patient rehabilitation units, supported accommodation services and community rehabilitation teams. Around 80% of people admitted to in-patient rehabilitation units are referred from acute in-patient units, with the remainder arriving from forensic in-patient settings.^4,5^ In England, most people admitted to an in-patient rehabilitation unit will have been in contact with mental health services for over a decade (median 13 years, interquartile range (IQR) 6–22) and have experienced recurrent in-patient admissions (median 4 admissions, IQR 2–9).^4,5^ These units are staffed by a multidisciplinary team, including psychiatrists, psychologists, occupational therapists, nurses and support workers. They support people to gain or regain the skills and confidence to manage the everyday tasks needed for a successful community discharge, through a personalised biopsychosocial approach. The specific treatments offered may include optimisation of medication and management of associated side-effects, including, where appropriate, a trial of clozapine;^6^ management of physical health conditions; a range of activities that promote specific skills (e.g. cooking) and develop the person’s interests and social networks (e.g. music, arts, sports); and psychological interventions, including cognitive–behavioural therapy for psychosis and family work. This specialist approach takes time; the median length of admission in an in-patient rehabilitation unit in England is 8 months (IQR 4–19^4,5^), and thus in-patient rehabilitation is an expensive component of the mental healthcare system. Nevertheless, cohort studies suggest that around two-thirds of the people they treat achieve and sustain successful community discharge.^7,8^

The gold standard for testing the effectiveness of an intervention is a randomised controlled trial. However, since in-patient rehabilitation is recommended by national healthcare guidelines, it would be unethical to withhold this from a control group of people with complex psychosis. The studies that have investigated the effectiveness of in-patient rehabilitation have therefore been observational cohort studies, comparing in-patient service use before and after an in-patient rehabilitation admission. Such studies have been conducted in the UK,^5,9^ Canada^10^ and Australia,^11^ and have consistently reported a reduction in in-patient service use after the rehabilitation admission compared with before. However, these studies are limited by small sample sizes,^9^ relatively short before and after periods,^10,11^ and unique characteristics of the service studied – one of the studies evaluated a tertiary in-patient rehabilitation unit.^5^ The current study aimed to address these limitations by using routinely reported electronic healthcare records to compare in-patient service use before and after admission to a standard in-patient rehabilitation unit. We also aimed to investigate the characteristics of individuals who were and were not discharged successfully from the in-patient rehabilitation unit, to inform whether these services may be more effective for subgroups of patients. This study was completed as part of the lead author’s doctoral thesis.^12^

## Method

### Design and setting

This study used deidentified electronic healthcare records from the Camden and Islington NHS Foundation Trust Clinical Records Interactive Search (CRIS) Database. Camden and Islington NHS Foundation Trust provides a range of in-patient and community adult mental health services to residents of two inner-city London boroughs. It has well-established rehabilitation services, comprising in-patient rehabilitation units, supported accommodation services and community rehabilitation teams. CRIS is a tool that deidentifies electronic healthcare records, providing approved researchers with a searchable database of structured and unstructured records.^13-15^ Structured records are any record that was created using a pre-determined drop-down options menu (e.g. for ethnicity) or a specific format (e.g. date for date of birth). Unstructured records are created using free text (e.g. a clinical note describing a healthcare contact or a general practitioner letter).

The cohort in this study was defined as any individual with a recorded admission to one of the Trust’s two high-dependency in-patient rehabilitation units (as defined by the Royal College of Psychiatrists’ typology of in-patient rehabilitation services^16^), where the admission started between 1 January 2010 and 30 April 2019 and lasted for a minimum of 84 days (which was considered a reasonable balance between individuals receiving an adequate dose of in-patient rehabilitation and having an adequate cohort size), and the individual had at least 365 days of records available before and after the admission. Therefore, the study utilised records pertaining to the period between 1 January 2009 and 30 April 2020.

### Data extraction

Study data were extracted from structured records, with structured in-patient service use data validated using free-text records (see ‘Data validation’ below for further detail). Primary diagnosis in this study was defined as the ICD-10 diagnosis that had a record date closest to the start date of the in-patient rehabilitation admission. Secondary, or comorbid, diagnoses were defined as any other diagnosis recorded during the study period.

For each in-patient admission, the start date, end date and admission type (acute, psychiatric intensive care, forensic or rehabilitation) were extracted from the structured records. Transfers between in-patient services (instances where an admission end date was contiguous with the admission start date of the next recorded admission) of the same type were coded as a continuous admission, whereas transfers to a different type of in-patient service were coded as a new admission.

### Data validation

Significant changes in an individual’s healthcare, such as an admission or discharge from an in-patient service, are recorded as a free-text record in the ‘progress notes’ section of the patient’s records. Free-text documents relating to in-patient admissions, such as discharge summaries, are also uploaded to the healthcare record system. The recording of in-patient admission start and end dates in free-text records provides a means of validating the admission start and end dates recorded in structured records that were extracted for this study.

We carried out two validations. One of the validations was of instances where the structured records indicated that the individual was admitted to the in-patient rehabilitation unit directly from the community rather than from another in-patient service (i.e. the start date of the in-patient rehabilitation admission did not match the end date of another in-patient admission). This validation was carried out because most referrals to in-patient rehabilitation units are from another type of in-patient service. To do this, unstructured records relating to these individuals recorded around the start date of the in-patient rehabilitation admission were reviewed to clarify where they were before they were admitted to the rehabilitation unit.

The other validation comprised a check of start and end dates of in-patient admissions for a randomly selected 10% of individuals in the study cohort, by reviewing the relevant unstructured records. This validation was completed to check on accuracy of the structure records for in-patient admission start and end dates used in this study. It was agreed that if more than 5% of the dates checked mismatched by more than a day, the admission dates of the whole cohort would be checked.

### Health of Nation Outcome Scales data

Data from clinical assessments made using the Health of Nation Outcomes Scale (HoNOS)^17^ were also extracted. The HoNOS is a clinician-rated clinical and social functioning assessment scale with good psychometric properties, which is used nationally and internationally.^18-20^ It consists of 12 items (1. aggression and overactivity, 2. self-harm, 3. problem drinking and drugs, 4. cognitive impairment, 5. physical impairment, 6. hallucinations and delusions, 7. depressed mood, 8. other mental health problem, 9. relationship problems, 10. daily living skills, 11. living conditions and 12. occupation/activities), each rated from 0 to 4, with a score of 0 indicating that there is no problem in this area affecting health or functioning and a score of 4 indicating a very severe problem. The HoNOS is recorded routinely by National Health Service (NHS) staff at admission and discharge from in-patient and community care. HoNOS assessments were extracted at two time-points: within 3 months of the start date and within 3 months of the end date of the rehabilitation admission.

### Analysis plan

Data analyses were carried out with Stata version 16.0 for Windows (StataCorp, College Station, Texas, USA; see https://www.stata.com/). The main analysis compared the number of in-patient days before and after the rehabilitation admission, using paired *t*-tests and negative binomial regression models.

The first *t*-test compared the number of in-patient days 1 year before the rehabilitation admission with 1 year after the rehabilitation admission. Further paired *t*-tests were conducted with longer before and after periods at yearly intervals, until there were an insufficient number of individuals for the comparison to be made.

Unlike the *t*-tests, all of the available before and after data were used in the negative binomial regression models. To account for the variance within and between individuals in the period of records before and after the rehabilitation admission, in-patient days were entered as the response variable and the period before and after the rehabilitation admission was entered as the exposure variable, with a binary time variable added to the model (pre-rehabilitation admission or post-rehabilitation admission).^21^

Two negative binomial regression models were planned: an unadjusted model and a model adjusted for potential confounder variables. Sociodemographic and clinical variables available in the CRIS Database, which, based on previous research^8^ and the authors’ clinical knowledge, may affect in-patient service use were added as potential confounder variables to the adjusted model. These were age at start of the rehabilitation admission; gender; ethnicity (White or Black, Asian and minority ethnicity); any recorded mental or physical health comorbidity; length of the rehabilitation admission (days); year the rehabilitation admission started (as a proxy for any change in the mental health system over time); whether they were admitted from a forensic unit (yes/no); whether they were discharged under a community treatment order (yes/no) and HoNOS ratings at the end of the rehabilitation admission for domains 6 (psychotic symptoms), 9 (relationships) and 10 (activities of daily living).

An estimate of the cost of in-patient service use 1, 2, 3 and 4 years before and after the in-patient rehabilitation admission was calculated by multiplying the number of in-patient days during each period by the NHS reference costs for the daily cost of an NHS mental health bed in 2021 (i.e. £428).^22^ A similar pre- and post-rehabilitation cost comparison was conducted by Bunyan et al.^9^ In a separate analysis, we estimated in-patient service use costs at 1, 2, 3 and 4 years before and after the rehabilitation admission, but this time we accounted for the cost of the rehabilitation admission. In this analysis, the ‘before’ periods included an estimate of what each individual’s in-patient service use and associated costs would have been during the period of their rehabilitation admission if they had not been admitted to the rehabilitation unit, based on the rate of their in-patient service use before the rehabilitation admission and the length of their rehabilitation admission. For example, if someone had 250 in-patient days over a period of 500 days before their rehabilitation admission (an in-patient service use rate of 0.5) and their rehabilitation admission lasted 200 days, their in-patient service use would be estimated as 100 days during the period of their rehabilitation admission. This assumes that their rate of in-patient service use before the rehabilitation admission would have remained stable if they had not had a rehabilitation admission. The ‘after’ costs included the cost of their actual rehabilitation admission. For the estimates in this second analysis, we used the local Camden and Islington NHS Foundation Trust acute and rehabilitation in-patient bed day costs provided by the Trust’s finance team for 2021 (£547 and £498, respectively) as there are no standard NHS costs specifically for acute or rehabilitation in-patient bed days.

Further analyses were conducted to investigate the sociodemographic and clinical characteristics of people who were and were not successfully discharged. Successful discharge was defined as discharge from the in-patient rehabilitation unit to the community without any readmission within 12 months of discharge. The sociodemographic and clinical characteristics of the successfully discharged group were compared with those that were not successfully discharged, using chi-squared tests and *t*-tests.

### Ethics and consent statement

The Camden and Islington NHS Foundation Trust CRIS Database has been granted ethical approval to be used in epidemiological research and service improvement (National Research Ethics Service Committee East of England – Cambridge Central: reference number 19/EE/0210), without the need to obtain informed consent of individuals given that the database contains only healthcare records of deidentified individuals. Approval for this project was granted by the Camden and Islington NHS Foundation Trust CRIS Database Oversight Committee on 28 February 2020.

## Results

### Sociodemographic and clinical characteristics

A total of 271 individuals were admitted to one of the two in-patient rehabilitation units during the study period (1 January 2010 and 30 April 2019). However, 99 of these individuals did not meet the eligibility criteria, most commonly (95/99) because they had fewer than 365 days of records available before and/or after their rehabilitation admission. A further four individuals were excluded as their in-patient rehabilitation admission was shorter than 84 days (7 of the 99 excluded individuals failed to meet both criteria). Therefore, 172 individuals were included in the study. Table 1 shows their sociodemographic and clinical characteristics. They had a mean age of 44 years (s.d. 14) and the majority were male (*n* = 101, 59%), White (*n* = 98, 57%) and single (*n* = 155, 91%). Almost three-quarters had a primary mental health ICD-10 diagnosis of schizophrenia (*n* = 126, 73%), around half had at least one mental or physical health comorbidity (*n* = 97, 56%) and around a third (*n* = 55, 32%) had multiple comorbidities. Sixteen individuals (9%) died during the study period, at a mean age of 65 years (s.d. 12).

**Table 1:** Sociodemographic and clinical characteristics (*N* = 172)

Characteristic	*n*	%
Age at the start date of the in-patient rehabilitation admission, *n* =172 (mean and s.d.)	44.2	13.8
Male	101	59
Female	71	41
Ethnicity, *n* = 172		
White British/Irish/other	98	57
Black	51	30
Mixed or other ethnicity	14	8
Asian	9	5
Marital status, *n* = 171		
Single (unmarried or without a civil partner)	155	91
Divorced/separated/widowed	10	6
Married/civil partner	6	4
Primary mental health ICD-10 diagnosis, *n* = 172^a^		
Schizophrenia disorder (F20–F24 and F26–F29)	126	73
Schizoaffective disorder (F25)	28	16
Manic episode (F30) or bipolar affective disorder (F31)	11	6
Other mental health disorder	7	4
Comorbid mental health diagnosis, *n* = 172^b^		
Substance misuse disorders (F10–F19)	44	26
Depression and anxiety disorders (F32–F48)	10	6
Disorders of adult personality and behaviour (F60–F69)	15	9
Comorbid physical health diagnosis, *n* = 172^b^		
Endocrine, nutritional and metabolic diseases (E00–E90)	41	24
Diseases of the nervous system (G00–G99)	8	5
Diseases of the circulatory system (I00–I99)	21	12
Diseases of the respiratory system (J00–J99)	10	6
Any mental or physical health comorbidity, *n* = 172	97	56
Multiple comorbidities, *n* = 172	55	32

a The ICD-10 primary mental health diagnosis recorded closest to the rehabilitation admission start date.

b Whether this ICD-10 diagnosis has ever been recorded during the study period (1 January 2009 to 30 April 2020) for this individual, in addition to the ‘Primary mental health ICD-10 diagnosis’.

### Data validation

Of the 172 individuals in the cohort, 50 (29%) did not have an admission recorded immediately before their rehabilitation admission in their structured records, suggesting they were referred to the in-patient rehabilitation unit from the community. However, the free-text records for these individuals showed that 39 of the 50 (78%) had actually been transferred from another in-patient unit. Most of these transfers (*n* = 23, 59%) were from another healthcare provider, which may explain why their admission data was missing from the structured records in the CRIS Database.

Validation of start and end dates of in-patient admissions recorded in the structured fields was conducted for 18 individuals (10% of the total individuals included in this study) for whom 211 admissions were recorded. Of the 422 start and end dates that were validated, 351 (83%) matched exactly and 409 (97%) matched within 1 day.

### Referral source and discharge destination

Supplementary Table 1 (available at https://doi.org/10.1192/bjo.2025.31) shows where individuals were before and after their rehabilitation admission (i.e. the referral source and discharge destination). The vast majority were transferred from another in-patient unit (*n* = 161, 94%). Three-quarters were discharged to the community (*n* = 130, 76%), and the remainder were transferred to another in-patient unit. Just over half (56%) were made subject to a community treatment order at discharge from the rehabilitation admission (*n* = 96, 74% of those discharged to the community).

### HoNOS scores

Supplementary Table 2 shows the HoNOS scores for each HoNOS item at the start and end of the rehabilitation admission. There was a high rate of missing data at both the start and end of the rehabilitation admission, with total HoNOS scores missing for 75 (44%) and 71 (41%) individuals, respectively. The total score was higher at the start of the admission (mean 33.1, s.d. 14.8) compared with the end of the admission (mean 27.5, s.d. 13.2). The three HoNOS items selected as confounder variables for the regression model (items 6, 9 and 10) received a score of 3 or 4 more often than the other HoNOS items across both time points, providing corroboration for our decision to include these three items in our regression model.

### In-patient service use before and after the rehabilitation admission

The median length of the rehabilitation admission was 318 days (IQR 191–455). Supplementary Table 3 shows the calendar year in which the rehabilitation admission started. Table 2 shows the period of records available before and after the rehabilitation admission, and in-patient service use during these periods. The median period that records were available before and after the rehabilitation admission was 4.1 years (IQR 2.6–6.2) and 5.4 years (IQR 3.1–7.0), respectively. The median percentage of days spent as an in-patient before the rehabilitation admission was 29% (IQR 18–52%), and after rehabilitation it was 8% (IQR 0–31%). Fewer than five individuals had no in-patient service use before their rehabilitation admission (<3%, exact number supressed to prevent the identification of individuals), whereas over a third had no in-patient service use after their rehabilitation admission (*n* = 64, 37%).

**Table 2:** In-patient service use before and after the rehabilitation admission (*N* = 172)

In-patient service use	Pre-rehabilitation admission		Post-rehabilitation admission	
	Mean (s.d.)	Median (IQR)	Mean (s.d.)	Median (IQR)
Duration of records, in years^a^	4.4 (2.2)	4.1 (2.6–6.2)	5.2 (2.4)	5.4 (3.1–7.0)
In-patient days	519 (400)	420 (262–653)	411 (595)	153 (0–583)
Number of admissions	3.8 (3.0)	3 (1.5–5)	1.9 (2.4)	1 (0–3)
Percentage of days spent as an in-patient	40% (31%)	29% (18–52%)	20% (26%)	8% (0–31%)

IQR, interquartile range.

a The start date used to calculate the pre-rehabilitation admission period was the date their first progress note is recorded or on the start date of their first recorded admission, whichever comes first within the study period (1 January 2009 to 30 April 2020). The end date used to calculate the post-rehabilitation admission period was the date records are available up until (30 April 2020) or their date of death if one is recorded.

Table 3 shows the results comparing the number of in-patient days during the 1-, 2-, 3- and 4-year periods before and after the in-patient rehabilitation admission. The 5-year comparison was not carried out as too few individuals in the cohort had this length of records before and after the rehabilitation admission. Although the cohort size decreased substantially with each additional year used in the comparison, each comparison consistently showed a statistically significant reduction in the number of in-patient days after the rehabilitation admission compared with before.

**Table 3:** In-patient days before and after the rehabilitation admission, by number of years of pre-post rehabilitation (*N* = 172)

Pre- and post-rehabilitation period	In-patient days pre-rehabilitation admission		In-patient days post-rehabilitation admission		Mean difference (95% CI)
	Mean	s.d.	Mean	s.d.	
One year	223.3	115.5	103.7	137.6	119.6 (95.8–143.4)
Two years, *n* = 123	354.6	203.4	185.5	248.6	169.1 (114.4–223.9)
Three years, *n* = 82	424.3	307.7	253.5	332.1	170.7 (64.9–276.6)
Four years, *n* = 36	561.4	447.5	305.6	420.2	255.8 (32.0–479.5)

In the unadjusted negative binomial regression model, the incidence rate ratio (IRR) comparing the period after the rehabilitation admission with the period before was 0.504 (95% CI 0.358–0.710). This shows that in-patient service use reduced by 50% after an in-patient rehabilitation admission compared with the period before.

The IRR increased slightly to 0.555 (95% CI 0.351–0.877) when adjusted for potential confounding variables. However, because of the missing HoNOS scores, the adjusted model did not include the full cohort (*n* = 100). Therefore, a *post-hoc* regression analysis was conducted that included the full cohort (*N* = 172), which adjusted for the same potential confounding variables except for the three HoNOS items, and this produced an IRR of 0.520 (95% CI 0.367–0.737).

### Cost of in-patient service use before and after the rehabilitation admission

The mean estimated cost of in-patient service use for the 1-year period before the rehabilitation admission was £95 585.84 (s.d. £49 417.89) and £44 392.56 (s.d. £58 894.49) for the 1-year period after the rehabilitation admission. Table 4 shows the estimated costs of in-patient service use for the 2, 3 and 4 years before and after the rehabilitation admission, which all showed a similar pattern of reduced costs after rehabilitation compared with before rehabilitation.

**Table 4:** In-patient service use costs before and after the in-patient rehabilitation admission (*N* = 172)

Pre- and post-rehabilitation period	In-patient costs pre-rehabilitation admission, £		In-patient costs post-rehabilitation admission, £	
	Mean	s.d.	Mean	s.d.
One year	95 585.84	49 417.89	44 392.56	58 894.49
Two years, *n* = 123	151 786.90	87 054.23	79 402.70	106 402.50
Three years, *n* = 82	181 581.60	131 683.20	108 508.40	142 141.50
Four years, *n* = 36	240 262.60	191 514.00	130 801.60	179 861.70

National Health Service (NHS) reference costs for the daily cost of an NHS mental health bed in 2021 (i.e. £428).^23^

Table 5 shows the before and after estimated in-patient service use costs for the same periods as reported in Table 4, but where the cost of the rehabilitation admission has been included as described earlier in the Method. These analyses showed the mean in-patient service use cost for the 1-year period before the rehabilitation admission was £205 477.60 (s.d. £144 431.40), and the mean in-patient costs for the 1-year period after the rehabilitation admission was slightly higher at £233 070.80 (s.d. £129 115.50). The estimated in-patient costs for the 2- and 3-year before and after periods were similar. However, the in-patient cost estimate in the 4 years after a rehabilitation admission was lower than the 4 years before (£316 168.10 (s.d. £227 626.20) *v*. £378 478.80 (s.d. £322 519.10)).

**Table 5:** In-patient service use costs before and after the in-patient rehabilitation admission, including the cost of the rehabilitation admission (*N* = 172)

Pre- and post-rehabilitation period	In-patient costs pre-rehabilitation admission, including estimated cost of in-patient service use during the rehabilitation admission, £^a^		In-patient costs post-rehabilitation admission, including cost of the rehabilitation admission itself, £^b^	
	Mean	s.d.	Mean	s.d.
One year	205 447.60	144 431.40	233 070.80	129 115.50
Two years, *n* = 123	264 714.20	176 887.60	269 010.10	158 205.70
Three years, *n* = 82	292 162.80	226 632.80	289 656.90	190 968.30
Four years, *n* = 36	378 478.80	322 519.10	316 168.10	227 626.20

a Includes an estimation of the cost of in-patient service use during the rehabilitation admission if there was not a rehabilitation admission. This cost is calculated using the individual’s proportion of days spent in an in-patient service before the rehabilitation admission and the length of their in-patient rehabilitation admission, and the cost per day for an acute mental health bed at Camden and Islington National Health Service (NHS) Foundation Trust (£547).

b Includes the cost of the in-patient rehabilitation admissions itself. This cost is calculated using the length of the in-patient rehabilitation admission, and the cost per day for a mental health rehabilitation bed at Camden and Islington NHS Foundation Trust (£498).

### Sociodemographic and clinical characteristics of individuals with and without a successful discharge

Overall, 89 (52%) individuals had a successful discharge which meant that they were discharged from the in-patient rehabilitation unit to the community and were not readmitted within 12 months. The remaining 83 (48%) individuals without a successful discharge were either discharged from the rehabilitation unit to another type of in-patient service (*n* = 42, of whom 26 were discharged to an acute or psychiatric intensive care unit, and 16 were discharged to a longer-term rehabilitation unit or other type of ward), or they were discharged to the community and readmitted within 12 months (*n* = 41). Those who were not successfully discharged were more likely to be Black than any other ethnicity (37 *v*. 22%; *P* = 0.033), and more likely to have a comorbid health condition (67 *v*. 46%; *P* = 0.005). They also had a larger proportion of days as an in-patient before (mean 46% (s.d. 31%) *v*. 34% (s.d. 30%); *P* = 0.011) and after the rehabilitation admission compared with the successfully discharged group (mean 38% (s.d. 27%) *v*. 3% (s.d. 8%); *P* < 0.001). Among those who were successfully discharged, 64 (72%) individuals had no further recorded in-patient service use after their in-patient rehabilitation admission.

## Discussion

This study found that following admission to an in-patient rehabilitation unit, the rate of subsequent in-patient service use was halved compared with the period before the rehabilitation admission. Adjusting for potential confounding variables had minimal impact on this estimate. Although this finding is consistent with those of other ‘before and after’ studies of in-patient rehabilitation services,^5, 9-11^ this study has a number of strengths that suggest it provides more robust evidence for the effectiveness of these services.

Bunyan et al^9^ compared in-patient service use 2 years before and after an in-patient rehabilitation admission in South London, but included only 22 individuals. In Canada, Awara et al^10^ compared in-patient service use 6 months before and after a rehabilitation admission for 53 individuals. In Australia, Parker et al^11^ compared in-patient service use for a large cohort (*N* = 501) of patients 1 year before and after admission to a community rehabilitation unit. Casetta et al^5^ compared in-patient service use for 147 individuals 2 years before and after admission to the National Psychosis Unit. All of these studies showed statistically significant reductions in in-patient service use following the rehabilitation admission. The current study included a relatively large cohort of individuals (*N* = 172) who were studied over a longer period before (mean 4.4 years, s.d. 2.2 years) and after (mean 5.2 years, s.d. 2.4 years) the rehabilitation admission than previous studies. Our analysis also took account of potential confounders, unlike previous studies. In addition, Casetta et al’s study^5^ focused on a specialist national service, whereas the rehabilitation units included in the current study only accept referrals from the catchment area of the local mental health NHS Trust within which they operate. This difference is of clinical relevance as in-patient rehabilitation units form part of a local rehabilitation pathway,^3^ and developing partnerships with local organisations (such as supported accommodation services, and educational and employment services) are key in enabling recovery and community engagement for the individuals with whom they work.

Our finding that in-patient service use is halved after a rehabilitation admission suggests that in-patient rehabilitation facilitates long-term stability, significantly reducing the chance of relapse and readmission. Over three-quarters of the cohort were discharged to the community at the end of their rehabilitation admission and over a third had no subsequent admissions. These positive outcomes (being discharged to the community and not being readmitted) were associated with having spent less time as an in-patient before the rehabilitation admission. This supports NICE’s recommendation^3^ that people with complex psychosis experiencing recurrent or lengthy admissions should be referred for rehabilitation much sooner, and after fewer acute in-patient admissions.

The extent of the reduction in in-patient service use after an in-patient rehabilitation admission is striking, given the very high level of in-patient service use for the present cohort. The present cohort spent a median of 29% (IQR 18–52%) as an in-patient during the pre-rehabilitation admission period, the equivalent of 3.5 months in a year. This is very high compared with the general level of in-patient service use for people with psychosis. For comparison, a study that looked at in-patient service use for 2147 people who presented with psychosis to a South London NHS Trust between 2007 and 2010, had a median of six (IQR 0–69 days) psychiatric in-patient days during the 5 years following their presentation.^23^ This comparison adds even greater weight to NICE’s recommendation that people with complex psychosis should be identified and referred to rehabilitation much sooner than what is currently happening.

We also found patients who were Black or had a comorbid health condition were less likely to be successfully discharged from their rehabilitation admission. These findings are helpful in identifying those who may benefit most from in-patient rehabilitation currently, and raise questions as to how rehabilitation services can tailor their interventions to support all patients more effectively. The issue of racial disparity in healthcare outcomes is not confined to rehabilitation services. In England, Black people with psychosis are three times more likely to be detained involuntarily in hospital compared with White people with psychosis.^24^ A meta-analysis using international data found that Black African and Black Caribbean patients were twice as likely to have a compulsory mental health admission, and twice as likely to be readmitted, than White patients.^25^

Although having a comorbid health condition does understandably complicate treatment, this constitutes the majority of people admitted to in-patient rehabilitation units. The urgent need to improve outcomes for people with multiple health conditions is recognised in national and international guidelines, which recommend addressing this need through greater integration and collaboration between mental and physical healthcare systems,^3,26,27^ including the NICE guideline for rehabilitation.^3^

Although this study indicates there is a substantial reduction in in-patient service use after an in-patient rehabilitation admission, there was considerable variation between individuals. As well as the characteristics discussed above, further work is needed to identify whether other characteristics (not examined in this study) predict the risk of relapse and readmission (e.g. psychiatric symptoms, social functioning, substance misuse, risk behaviours). This would assist in the development of individualised relapse prevention plans and may help reduce readmissions.

Unsurprisingly, as they are based on the same data, our cost estimate of in-patient service use before and after the rehabilitation admission is consistent with our comparison for in-patient days: in-patient service use costs were lower after the rehabilitation admission compared with the period before. However, when the cost of the rehabilitation admission itself is also considered, this difference only appears in the 4-year comparison. This result should be interpreted with caution as the size of the cohort is much smaller for the 4-year comparison than it is for the other shorter before and after comparisons. The standard deviation for each of the mean estimates across all of the comparison are also quite large.

Furthermore, the cost estimates were limited to in-patient service use, and other health and social care costs, such as supported accommodation and community rehabilitation team input, were not included. Although more rigorous cost-effective analyses are required, these analyses nevertheless suggest that in-patient rehabilitation may provide a worthwhile investment; however, from a purely financial perspective, the return on the investment is likely to be achieved in the longer rather than the shorter term.

The value of in-patient rehabilitation should, of course, not be viewed only in terms of the financial benefits for the system, but also in terms of its effect on the individual. By the time a patient is admitted to an in-patient rehabilitation unit, they are likely to have been unwell for over a decade and to have had multiple admissions. At this stage, their confidence in being able to lead a meaningful life and participate in society is likely to be very low. Our finding that in-patient rehabilitation is associated with reduced subsequent in-patient service use strongly suggests that these services enable people’s recovery. The benefits of having adequate local in-patient rehabilitation are not limited to the people that directly use these services. Reducing ineffective and often extended acute in-patient admissions for this group frees these high-demand acute beds for others who are more likely to benefit from them.

### Limitations

We acknowledge limitations to our study. We used data from a single inner-city NHS Trust in London with a well-established local rehabilitation pathway, and findings may differ to areas with less well-established rehabilitation pathways. We did not include evaluation of how other components in the rehabilitation pathway, such as the availability and effectiveness of specialist supported accommodation services and community rehabilitation team input, may affect the effectiveness of in-patient rehabilitation services. A well-established pathway providing good community support is likely to help to reduce the need for subsequent readmission.

The study was also limited by the use of healthcare data that were not collected for the purpose of research. We did carry out validations of the in-patient service use data used in this study, but there may still be inaccuracies in the data used in this study. This is perhaps demonstrated by the high level of missing data for HoNOS assessments in the present study. It is standard practice for an HoNOS assessment to be completed at the start and end of a treatment, including an in-patient rehabilitation admission.^28^ However almost half of the individuals in this study did not have an HoNOS assessment recorded within 3 months of the start date of their rehabilitation admission, nor did they have an HoNOS assessment recorded within 3 months of their rehabilitation admission discharge date. It is unclear why there were so many individuals in this study without a recorded HoNOS assessment when such an assessment should have taken place, but it is illustrative of the potential issues in using routine healthcare data in research. In relation to this limitation regarding the available data for this study, in-patient service use as an outcome is a very narrow outcome for in-patient rehabilitation. Other important outcomes, such as measures of personal recovery, autonomy and social inclusion, should be considered in future research.

Almost everyone in our cohort had a primary diagnosis of schizophrenia, schizoaffective disorder or bipolar affective disorder, which is consistent with the other before and after studies.^5, 9-11^ That more than half of our cohort had at least one comorbid mental or physical health condition was also to be expected, given what is already known about this population.^3^ However, what was unexpected was the lack of autism spectrum disorder as a comorbid health condition, as the association between the two conditions is well evidenced.^29^ This may indicate that some diagnoses are underreported in the data-set used for this study, at least in the structured fields that were used. Other studies using similar data-sets have used natural language processing approaches to extract diagnoses from free-text records.^23^ Free-text records could have been used more than they were in the present study, to improve data quality and data availability.

Finally, because of the difficulties of randomisation and identification of a suitable comparison group, as with previous studies of in-patient rehabilitation,^5, 9-11^ this study was observational in design. Causality cannot therefore be inferred and ‘regression to the mean’ may explain the reduction in in-patient service use.^30^ However, this seems unlikely to be the full explanation, given the magnitude of the reduction in in-patient service use that we observed.

In conclusion, in-patient mental health rehabilitation services are designed to support people with complex psychosis to gain and regain skills that are essential to living in the community. These services are an important component of the rehabilitation pathway and should be available locally, as per NICE guidelines.^3^ Although in-patient rehabilitation services are an expensive component of the mental healthcare system, there has been a lack of research investigating their effectiveness. Our study partially addressed this and found that in-patient service use was reduced by half in the period after the rehabilitation admission compared with the period before the rehabilitation admission. The lack of a control group means that causality cannot be inferred, and further studies investigating other outcomes are needed.

## Supporting information

Dalton-Locke et al. supplementary material 1 Dalton-Locke et al. supplementary material

Dalton-Locke et al. supplementary material 2 Dalton-Locke et al. supplementary material

## Data Availability

The data that support the findings of this study are available to approved researchers through Camden & Islington National Health Service (NHS) Foundation Trust. The data are not publicly available due to restrictions on data access by the Data Controller in the interest of patient confidentiality.

## Supplementary material

The supplementary material can be found at https://doi.org/10.1192/bjo.2025.31
