# Supplementary material for: In-patient service use before and after a mental health in-patient rehabilitation admission
Source: BJPsych Open. 2025 Apr 1;11(3):e80. doi: 10.1192/bjo.2025.31 (PMC12052572; doi:10.1192/bjo.2025.31)
Supplement: Dalton-Locke et al. supplementary material 1 — Dalton-Locke et al. supplementary material [file S2056472425000316sup001.docx]

# Inpatient service use before and after a mental health inpatient rehabilitation admission:

# Supplementary tables

Supplementary table 1: Pre- and post-rehabilitation admission location, and Community Treatment Order status at rehabilitation admission discharge (N=172)

|  | **n** | **%** |
| --- | --- | --- |
| **Location immediately prior to the rehabilitation admission, n=172** | - | - |
| **Other inpatient service** | 161 | 94 |
| **Acute (including psychiatric intensive care unit)** | 133 | 83 |
| **Forensic** | 17 | 11 |
| **Other** | 11 | 7 |
| **Community** | 11 | 6 |
| **Location immediately after the rehabilitation admission, n=172** | - | - |
| **Other inpatient service** | 42 | 24 |
| **Community** | 130 | 76 |
| **Placed on Community Treatment Order at rehabilitation admission discharge, n=172** | 96 | 56 |

Supplementary table 2: HoNOS scores (N=172)

| **HoNOS at rehabilitation start date** | **Score (n (%))** | | | | |
| --- | --- | --- | --- | --- | --- |
| **Item** | **0** | **1** | **2** | **3** | **4** |
| **1. Aggression and overactivity, n=97** | 33 (34%) | 25 (26%) | 19 (20%) | 15 (15%) | 5 (5%) |
| **2. Self-harm, n=97** | 87 (90%) | 2 (2%) | 5 (5%) | 2 (2%) | 1 (1%) |
| **3. Problem drinking and drugs, n=97** | 59 (61%) | 10 (10%) | 11 (11%) | 6 (6%) | 11 (11%) |
| **4. Cognitive impairment, n=97** | 58 (60%) | 8 (8%) | 21 (22%) | 9 (9%) | 1 (1%) |
| **5. Physical impairment, n=97** | 51 (53%) | 16 (16%) | 24 (25%) | 5 (5%) | 1 (1%) |
| **6. Hallucinations and delusions, n=97** | 16 (16%) | 9 (9%) | 24 (25%) | 37 (38%) | 11 (11%) |
| **7. Depressed mood, n=96** | 44 (46%) | 24 (25%) | 18 (19%) | 9 (9%) | 1 (1%) |
| **8. Other mental health problem, n=96** | 41 (43%) | 9 (9%) | 18 (19%) | 21 (22%) | 7 (7%) |
| **9. Relationship problems, n=97** | 17 (18%) | 13 (13%) | 29 (30%) | 29 (30%) | 9 (9%) |
| **10. Daily living skills, n=97** | 14 (14%) | 5 (5%) | 35 (36%) | 31 (32%) | 12 (12%) |
| **11. Living conditions, n=97** | 46 (47%) | 9 (95) | 14 (14%) | 15 (15%) | 13 (13%) |
| **12. Occupation/activities, n=97** | 43 (44%) | 5 (5%) | 16 (165) | 22 (23%) | 11 (11%) |
| **Total standardised score, n=97 (mean, SD)*** | 33.1 | 14.8 | - | - | - |
| **HoNOS at rehabilitation end date** | **Score (n (%))** | | | | |
| **Item** | **0** | **1** | **2** | **3** | **4** |
| **1. Aggression and overactivity, n=101** | 40 (40%) | 31 (31%) | 18 (18%) | 8 (8%) | 4 (4%) |
| **2. Self-harm, n=101** | 91 (90%) | 7 (7%) | 2 (2%) | 0 (0%) | 1 (1%) |
| **3. Problem drinking and drugs, n=101** | 61 (60%) | 16 (16%) | 14 (14%) | 8 (8%) | 2 (2%) |
| **4. Cognitive impairment, n=101** | 55 (54%) | 23 (23%) | 16 (16%) | 7 (7%) | 0 (0%) |
| **5. Physical impairment, n=101** | 55 (54%) | 18 (18%) | 19 (19%) | 6 (6%) | 3 (3%) |
| **6. Hallucinations and delusions, n=101** | 21 (21%) | 9 (9%) | 38 (38%) | 23 (23%) | 10 (10%) |
| **7. Depressed mood, n=101** | 48 (48%) | 19 (19%) | 27 (27%) | 7 (7%) | 0 (0%) |
| **8. Other mental health problem, n=100** | 44 (44%) | 12 (12%) | 26 (26%) | 16 (16%) | 2 (2%) |
| **9. Relationship problems, n=101** | 24 (24%) | 12 (12%) | 25 (25%) | 39 (39%) | 1 (1%) |
| **10. Daily living skills, n=100** | 15 (15%) | 20 (20%) | 39 (39%) | 25 (25%) | 1 (1%) |
| **11. Living conditions, n=101** | 58 (57%) | 12 (12%) | 14 (14%) | 14 (14%) | 3 (3%) |
| **12. Occupation/activities, n=101** | 47 (47%) | 17 (17%) | 22 (22%) | 14 (14%) | 1 (1%) |
| **Total standardised score, n=101 (mean, SD)*** | 27.5 | 13.2 | - | - | - |
| *Total score is out of 100 and standardised so that assessments with 1, 2, or 3 missing items are comparable. HoNOS assessments with more than 3 items missing are treated as missing for total standardised score. | | | | | |

**Supplementary table 3: The calendar year in which the rehabilitation admission started (N=172)**

| **Calendar year** | **n (%)** |
| --- | --- |
| **2010, n=172** | 22 (13) |
| **2011, n=172** | 22 (13) |
| **2012, n=172** | 24 (14) |
| **2013, n=172** | 23 (13) |
| **2014, n=172** | 25 (15) |
| **2015, n=172** | 15 (9) |
| **2016, n=172** | 18 (10) |
| **2017, n=172** | 17 (10) |
| **2018, n=172** | 6 (3) |
| **2019, n=172** | 0 (0) |
