# Supplementary material for: In-patient service use before and after a mental health in-patient rehabilitation admission
Source: BJPsych Open. 2025 Apr 1;11(3):e80. doi: 10.1192/bjo.2025.31 (PMC12052572; doi:10.1192/bjo.2025.31)
Supplement: Dalton-Locke et al. supplementary material 2 — Dalton-Locke et al. supplementary material [file S2056472425000316sup002.pdf]

Inpatient service use before and after a mental health inpatient rehabilitation admission

Christian Dalton-Locke, Louise Marston, Justin Yang, David Osborn and Helen Killaspy

BJPsych Open (2025) 1–8. doi: 10.1192/bjo.2025.31

Supplementary tables

Supplementary table 1: Pre- and post-rehabilitation admission location, and Community Treatment Order status at rehabilitation admission discharge (N=172)

	n	%
**Location immediately prior to the rehabilitation admission, n=172**	-	-
**Other inpatient service**	161	94
**Acute (including psychiatric intensive care unit)**	133	83
**Forensic**	17	11
**Other**	11	7
**Community**	11	6
**Location immediately after the rehabilitation admission, n=172**	-	-
**Other inpatient service**	42	24
**Community**	130	76
**Placed on Community Treatment Order at rehabilitation admission discharge, n=172**	96	56

Supplementary table 2: HoNOS scores (N=172)

HoNOS at rehabilitation start date	Score (n (%))				
Item	0	1	2	3	4
1. Aggression and overactivity, n=97	33 (34%)	25 (26%)	19 (20%)	15 (15%)	5 (5%)
2. Self-harm, n=97	87 (90%)	2 (2%)	5 (5%)	2 (2%)	1 (1%)
3. Problem drinking and drugs, n=97	59 (61%)	10 (10%)	11 (11%)	6 (6%)	11 (11%)
4. Cognitive impairment, n=97	58 (60%)	8 (8%)	21 (22%)	9 (9%)	1 (1%)
5. Physical impairment, n=97	51 (53%)	16 (16%)	24 (25%)	5 (5%)	1 (1%)
6. Hallucinations and delusions, n=97	16 (16%)	9 (9%)	24 (25%)	37 (38%)	11 (11%)
7. Depressed mood, n=96	44 (46%)	24 (25%)	18 (19%)	9 (9%)	1 (1%)
8. Other mental health problem, n=96	41 (43%)	9 (9%)	18 (19%)	21 (22%)	7 (7%)
9. Relationship problems, n=97	17 (18%)	13 (13%)	29 (30%)	29 (30%)	9 (9%)
10. Daily living skills, n=97	14 (14%)	5 (5%)	35 (36%)	31 (32%)	12 (12%)
11. Living conditions, n=97	46 (47%)	9 (95)	14 (14%)	15 (15%)	13 (13%)
12. Occupation/activities, n=97	43 (44%)	5 (5%)	16 (165)	22 (23%)	11 (11%)
Total standardised score, n=97 (mean, SD)*	33.1	14.8	-	-	-
**HoNOS at rehabilitation end date**	**Score (n (%))**				
**Item**	**0**	**1**	**2**	**3**	**4**
1. Aggression and overactivity, n=101	40 (40%)	31 (31%)	18 (18%)	8 (8%)	4 (4%)
2. Self-harm, n=101	91 (90%)	7 (7%)	2 (2%)	0 (0%)	1 (1%)
3. Problem drinking and drugs, n=101	61 (60%)	16 (16%)	14 (14%)	8 (8%)	2 (2%)
4. Cognitive impairment, n=101	55 (54%)	23 (23%)	16 (16%)	7 (7%)	0 (0%)
5. Physical impairment, n=101	55 (54%)	18 (18%)	19 (19%)	6 (6%)	3 (3%)
6. Hallucinations and delusions, n=101	21 (21%)	9 (9%)	38 (38%)	23 (23%)	10 (10%)
7. Depressed mood, n=101	48 (48%)	19 (19%)	27 (27%)	7 (7%)	0 (0%)
8. Other mental health problem, n=100	44 (44%)	12 (12%)	26 (26%)	16 (16%)	2 (2%)
9. Relationship problems, n=101	24 (24%)	12 (12%)	25 (25%)	39 (39%)	1 (1%)
10. Daily living skills, n=100	15 (15%)	20 (20%)	39 (39%)	25 (25%)	1 (1%)
11. Living conditions, n=101	58 (57%)	12 (12%)	14 (14%)	14 (14%)	3 (3%)
12. Occupation/activities, n=101	47 (47%)	17 (17%)	22 (22%)	14 (14%)	1 (1%)
Total standardised score, n=101 (mean, SD)*	27.5	13.2	-	-	-

*Total score is out of 100 and standardised so that assessments with 1, 2, or 3 missing items are comparable. HoNOS assessments with more than 3 items missing are treated as missing for total standardised score.

Supplementary table 3: The calendar year in which the rehabilitation admission started (N=172)

Calendar year	n (%)
**2010, n=172**	22 (13)
**2011, n=172**	22 (13)
**2012, n=172**	24 (14)
**2013, n=172**	23 (13)
**2014, n=172**	25 (15)
**2015, n=172**	15 (9)
**2016, n=172**	18 (10)
**2017, n=172**	17 (10)
**2018, n=172**	6 (3)
**2019, n=172**	0 (0)
